# Supplementary material for: Bayesian networks elucidate complex genomic landscapes in cancer
Source: Commun Biol. 2022 Apr 4;5:306. doi: 10.1038/s42003-022-03243-w (PMC8980036; doi:10.1038/s42003-022-03243-w)
Supplement: Supplementary file 2 — Supplementary Information [file 42003_2022_3243_MOESM2_ESM.pdf]

SUPPLEMENTARY INFORMATION FOR COMMSBIO-21-2803A
Bayesian networks elucidate complex genomic landscapes in cancer

Nicos Angelopoulos[1,2,*] , Aikaterini Chatzipli[1], Jyoti Nangalia[1], Francesco Maura[3], Peter J. Campbell[1]

[1] *The Cancer, Ageing and Somatic Mutation Programme, Wellcome Sanger Institute, Hinxton, Cambridgeshire CB10 1SA, UK* [2] *Systems Immunity Research Institute, Medical School, Cardiff University, Cardiff, CF14 4XN, UK* [3] *Myeloma Program, Sylvester Comprehensive Cancer Center, University of Miami, Miami, FL, USA*

Supplementary Note 1: Software

Our software is written in the *SWI-Prolog* [4] programming environment with calls to *R* via *Real* [1], and to *Gobnilp* and *grapviz* via system calls. The core BN learning is via the *Gobnilp* software [2] which in turn depends on the *SCIP* optimization suite [3]. *R* is used for statistical testing, such as Fisher's test, multiple hypothesis correction and for heatmap construction. Visualization of networks is done via system calls to graphviz.

The overall control of the analysis is implemented in the package *gbn* which can be easily installed from within *SWI-Prolog*.

```
?- pack_install(gbn).
```

The library is also availabe on github: `https://github.com/nicos-angelopoulos/gbn`. Once the library has been loaded via:

```
?- library(gbn).
```

each BN described in this paper can be reconstructed by loading and executing simple querries of the form:

```
?- [pack('runs/gbns_in_cancer/aml')].
?- aml.
```

*To whom correspondence should be addressed. Email: angelopoulosn@cardiff.ac.uk.

The software includes functions (called predicates in *Prolog*) for running multiple experiments and producing all the different type of networks and statistical plots in this paper. The full list of prepared queries is: `aml`, mpn, mye, coa, gbm, ran as shown above.

Because of the large number of software dependencies, we also provide a complete OS (operating system) image for the Raspberry pi 4 architecture: `https://stoics.org.uk/~nicos/sware/gbn/gbn_image.html`. Once downloaded the image can be written into an SD card which can then used to boot a Raspberry 4 computer into an environment that included all necessary dependencies.

Supplementary Note 2: Parameter selection

Parameter μ controls the number of variables to be included in the network learning step. The main objective is to remove events (variables) which are infrequent in the dataset, as these are unlikely to play an important role in the constructed networks. The user defines a simple threshold in the form of an integer and any variable that corresponds to a genomic event that appears in less than μ samples is removed. Figure 1, shows the effect of μ (x-axis) on the number of variables that remain (y-axis) for the AML dataset (LHS), and the effect of μ on network density (y-axis) for the same dataset and $\epsilon = 7$ (RHS).

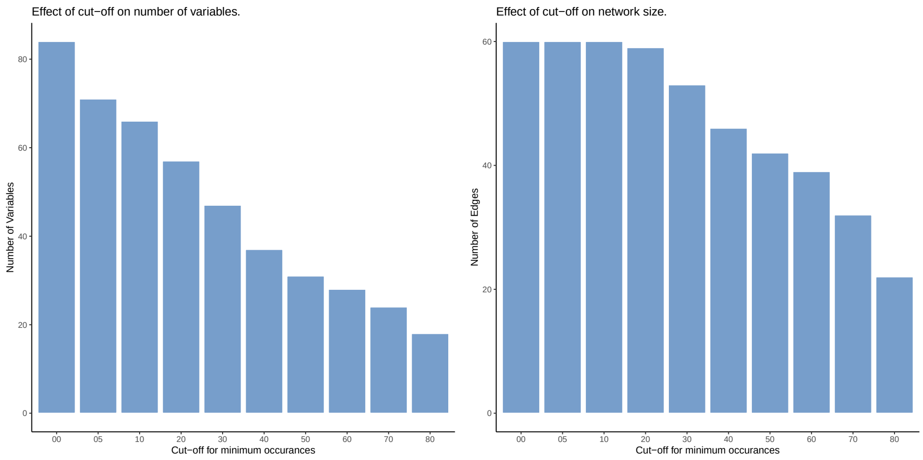

Supplementary Figure 1: Effect of μ on learning Bayesian networks in the AML dataset. (LHS) Varying μ (x-axis) against number of variables (y-axis) that will be included at that value of μ. (RHS) μ versus number of edges of the learnt BN ($\epsilon = 7$).

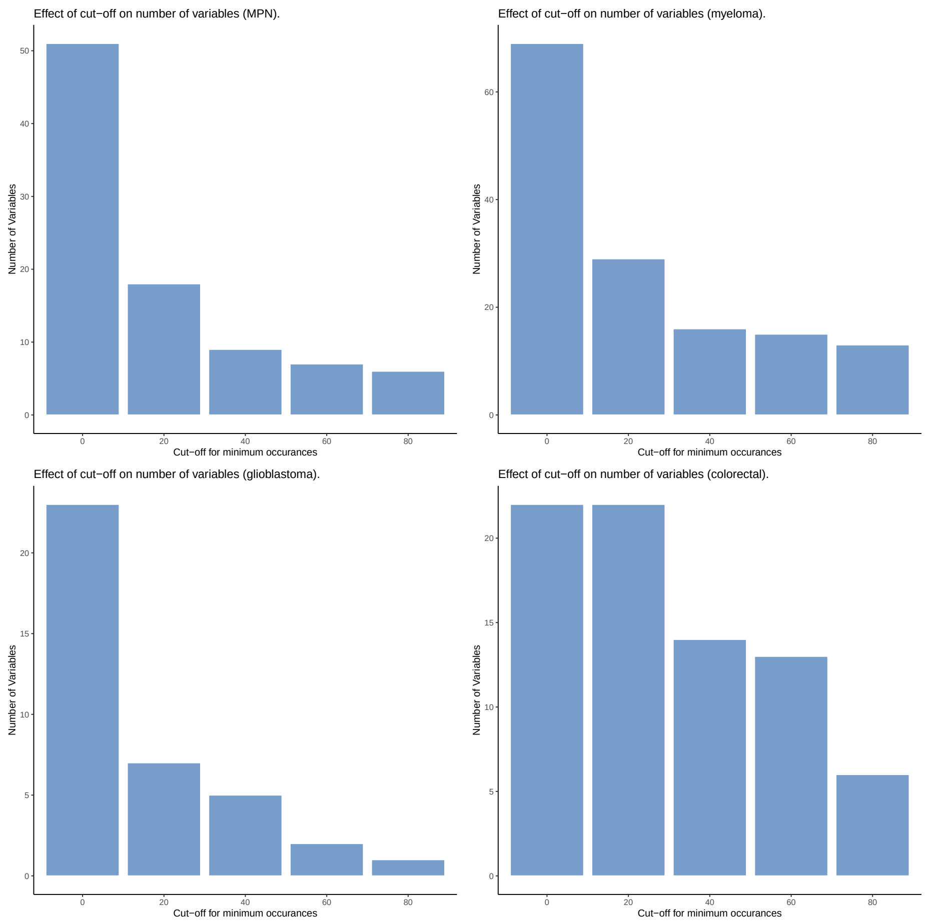

Supplementary Figure 2: Effect of μ on the number of variables for the four other datasets. From top left and travelling clockwise: MPN, myeloma, glioblastoma and colorectal.

Parameter ϵ is the *Gobnilp* parameter edge_penalty and is a single integer value typically in the range $1-20$. The higher the value the sparser the networks due to removal of the more weak edges. Although there is no formal guarantee for monotonicity, it is almost always the case that the networks for higher ϵ are subsets of those for lower values. This is certainly the case for all experiments we have ran for these datasets. Figure 3 shows how the number of edges for dataset AML varies (y-axis) as we change ϵ. Selecting the value for this parameter is usually straightforward. For datasets with few variables smaller values of ϵ might be more appropriate, whereas for larger number of variables greater ϵ values will assist will keeping the number of edges to the more important ones.

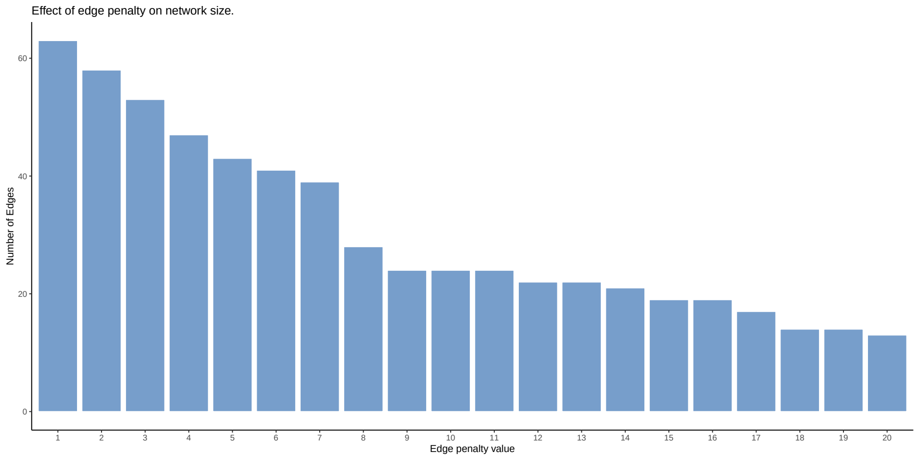

Supplementary Figure 3: Effect of ϵ on number of edges in the learnt BNs. For the AML dataset with $\mu = 60$ we ran 20 different learning experiments for varying ϵ and ploting this against the number of edges of the learnt BN.

Supplementary Note 3: Robustness

To quantify robustness of the networks with respect to ϵ we ran *Gobnilp* on each dataset ranging the value of ϵ from 1 to 10. These will create denser networks for ϵ value of 1 and sparser networks for the value 10. For each of the sparsest network of a dataset, we compare all other networks by counting the number of edges of the base (sparse) network that are also present on the denser ones. In all but one case all the edges were present (100%). Only in the case of myeloma there was a single edge $NRAS - del13q14$ that present in ϵ values $6 - 10$ and absent in the rest. The robustness measure for myeloma was thus 96.82%. In keeping with our removal of directionality of edges in BNs, here we also ignore directionality in counting presence of a link. As in the case of visualising the networks, our choice is justified by the fact that we do not perforrm interventional experiments and thus cannot establish causaility or direction. The overall measure of robustness was 99.36%. Please note that in the case of the glioblastoma because the network at ϵ has no edges we used the value of 5 as the base case.

Supplementary Note 4: Datasets

All datasets described in this paper are provided with our software (`data/gbns_in_cancer/`) in a format ready to be used as inputs to our scripts. They are also availabe on github: `https://github.com/nicos-angelopoulos/gbn/data/gbns\_in\_cancer`. Figure 4 and Figure 5 show the distribution of genomic events in AML. On the x-axis are the driver events and the heights of the bars show the number of patients in the cohort in which the specific event was

detected. Bars are colour coded with the number of total events. Light blue (labelled by 1 on the legend) shows the number of patients in which this event was the only one detected ($total = 1$). Based on the single event colour it can be seen that TET and DNMT3A are less likely to be a single event than their neighbours (event complex should be discounted when comparing to TET as it is a composite event).

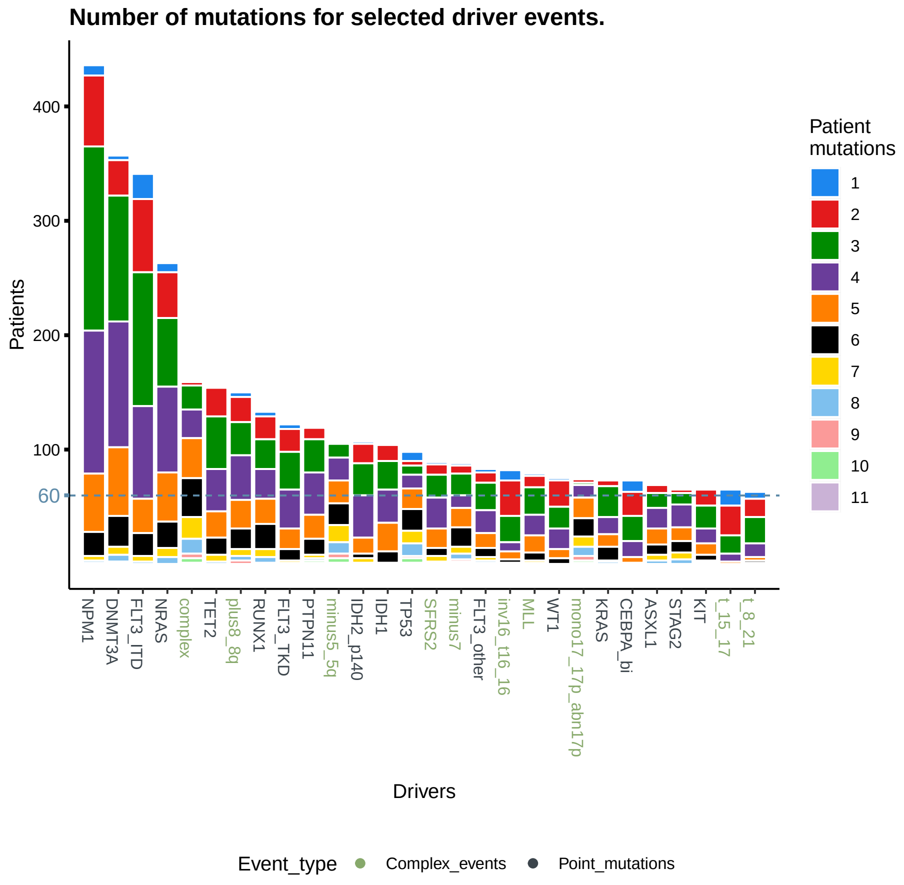

Supplementary Figure 4: Histogram of genomic driver events against patients in AML. This plot shows all selected drivers: those that appear on 60 or more patients. Each bar shows the number of patients for which the specific event (x-axis) was found by sequencing. Colours code for the number of total events in each patient. For instance, light blue (1) codes for the number of patients in which the specific event was the only driver event.

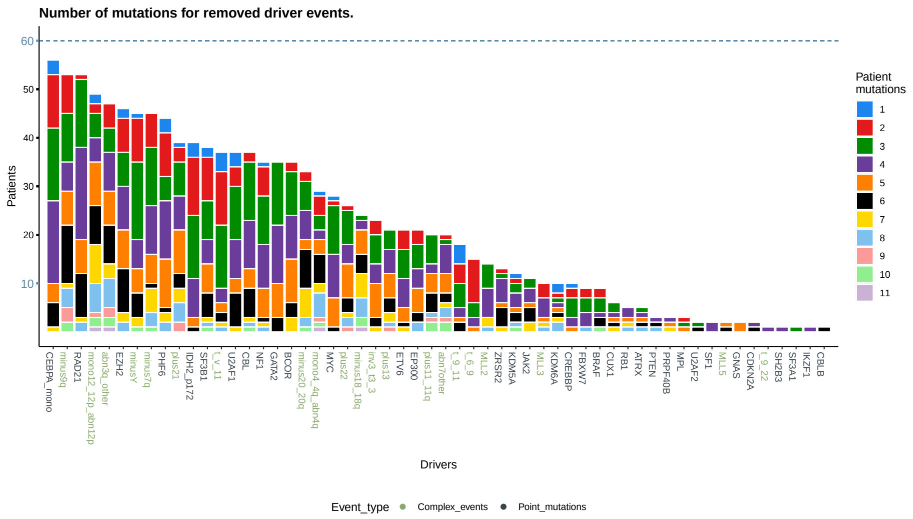

Supplementary Figure 5: Histogram of genomic driver events against patients in AML. This plot shows all removed drivers: those that appear on less than 60 patients. Each bar shows the number of patients for which the specific event (x-axis) was found by sequencing. Colours code for the number of total events in each patient. For instance, light blue (1) codes for the number of patients in which the specific event was the only driver event.

Supplementary Note 5: Additional networks

Here we provide additional BN and gated BN figures to complement all datasets analysed in this paper. For each dataset analysis its built BN and corresponding gated BN networks appear in either the main paper or in the supplement.

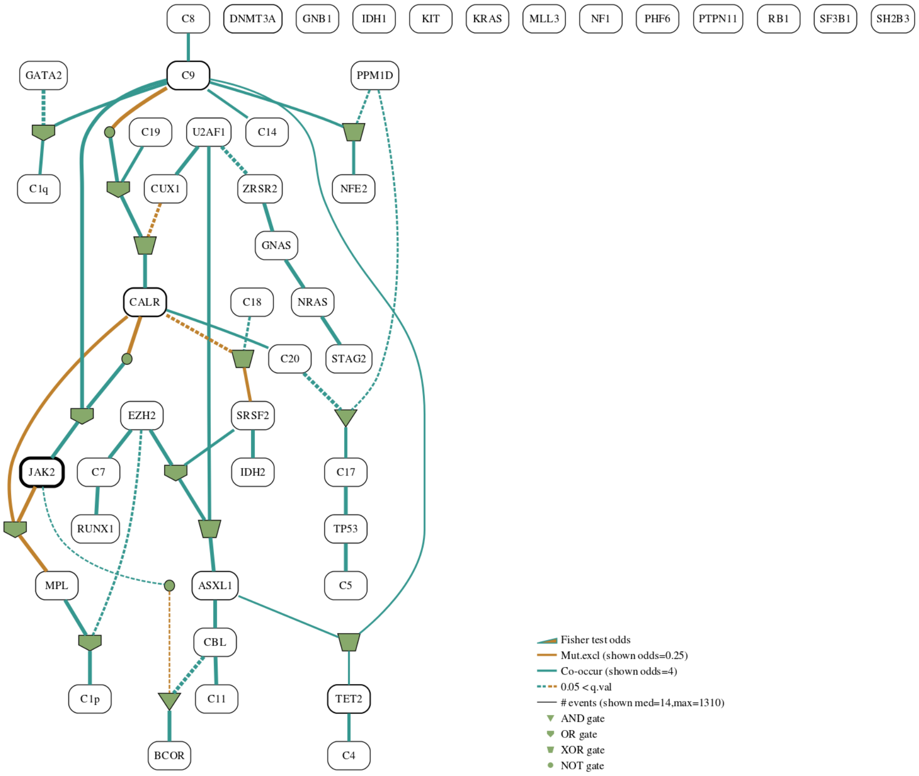

Supplementary Figure 6: Gated BN for MPN dataset ($\mu = 5, \epsilon = 3$).

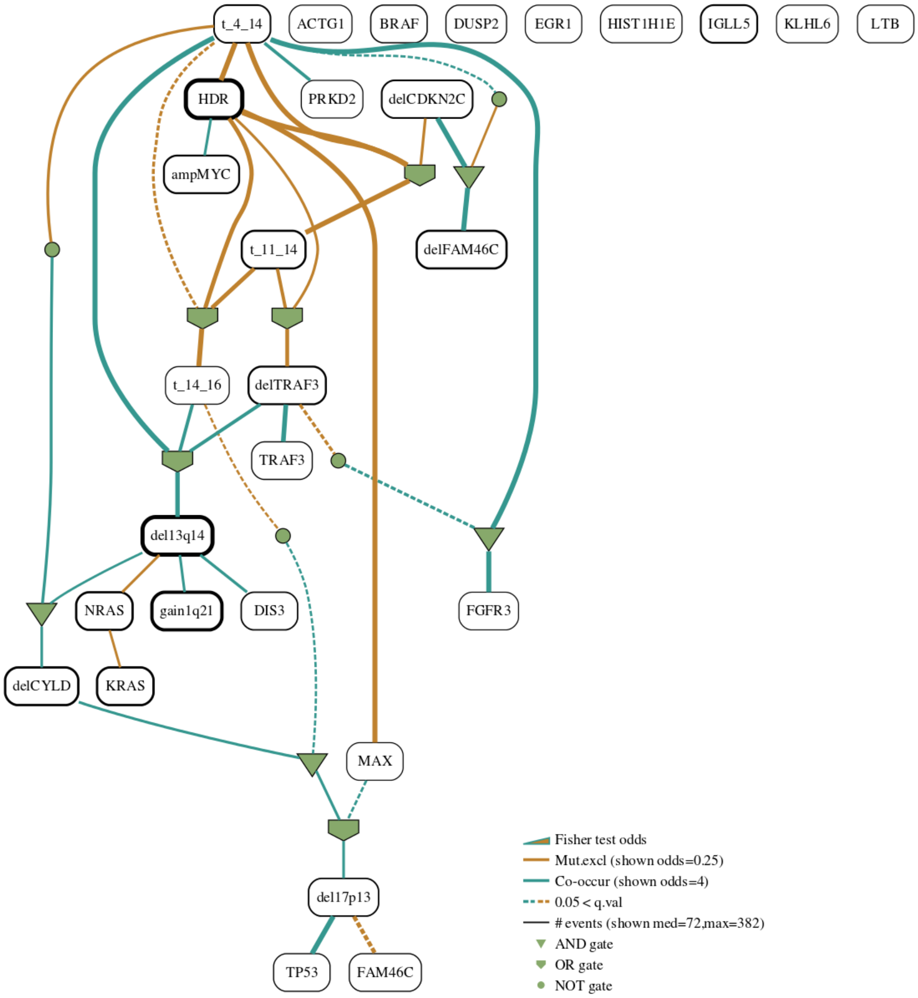

Supplementary Figure 7: Gated BN for myeloma dataset. Ran with same parameters as the non gated version ($\mu = 20, \epsilon = 2$).

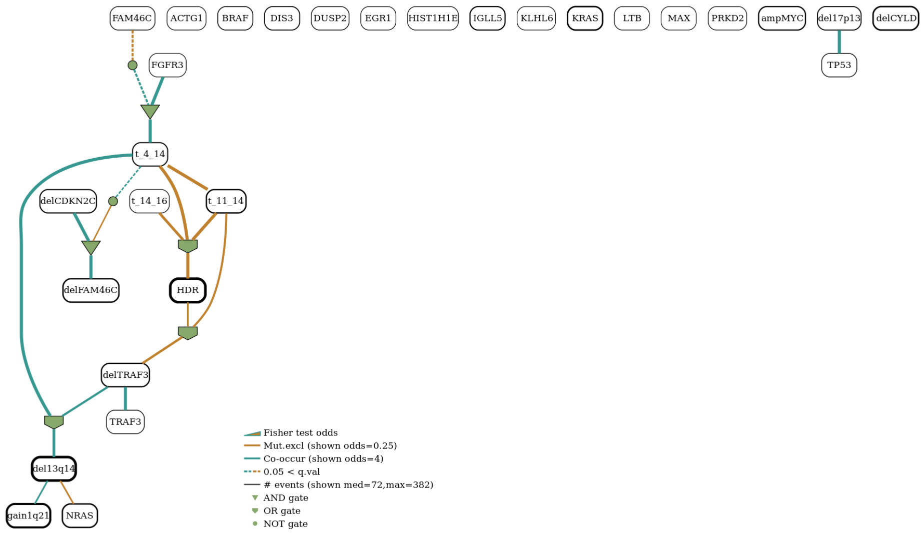

Supplementary Figure 8: Alternative myeloma gated BN. This is the minimum complexity containing the most important links ($\mu = 20, \epsilon = 12$).

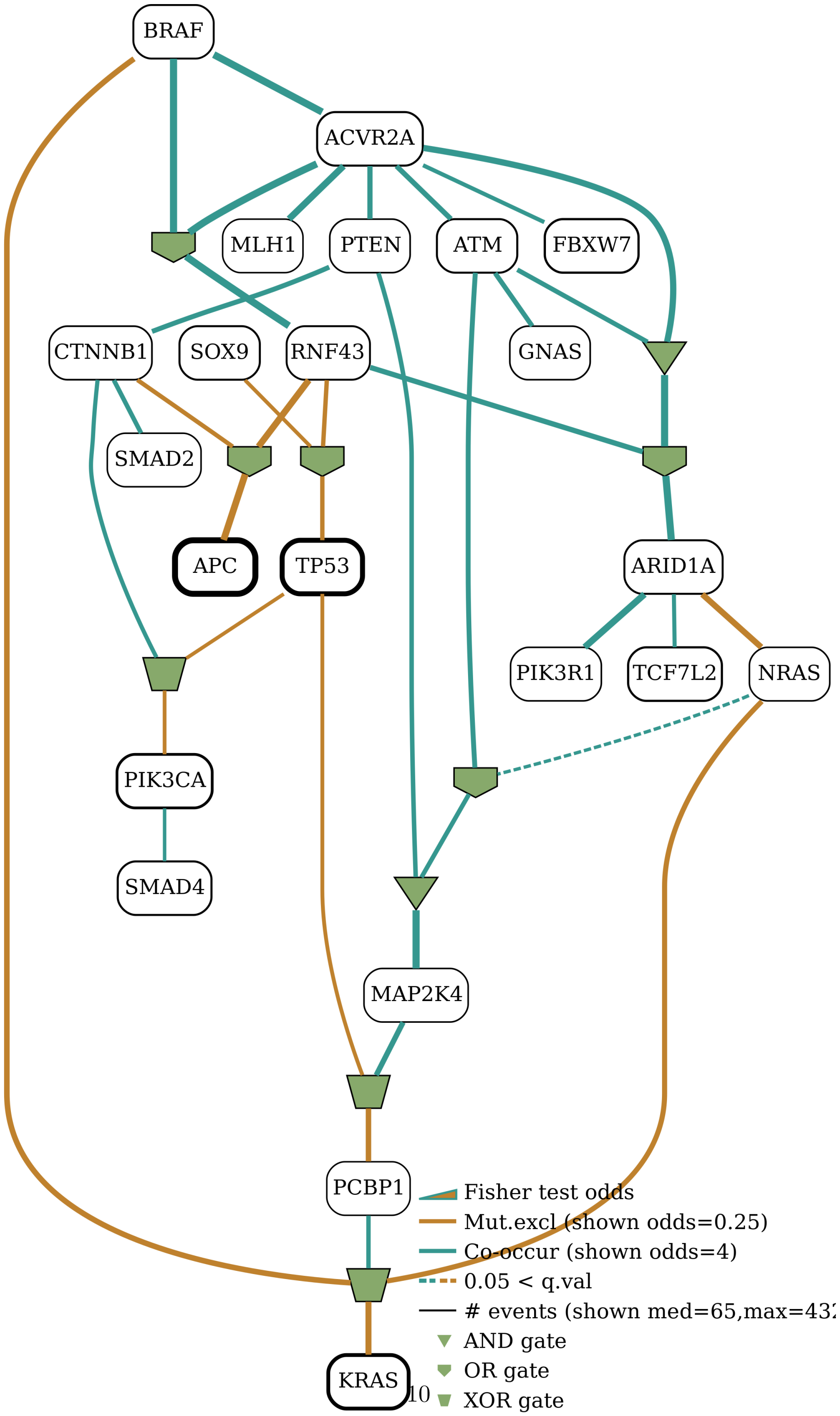

Supplementary Figure 9: Gated BN for colon adenocarcinoma dataset from TCGA (μ =5, ϵ =1).

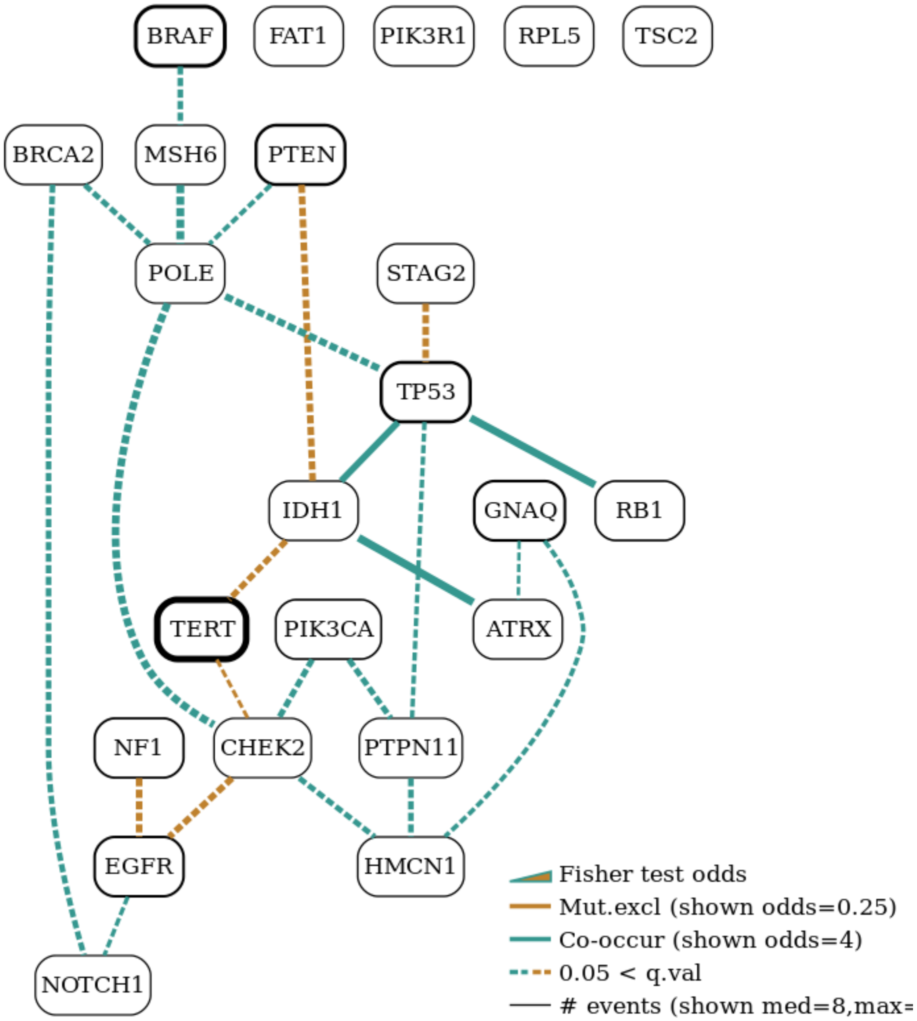

Supplementary Figure 10: BN for the glioblastoma dataset ($\mu = 5, \epsilon = 1$).

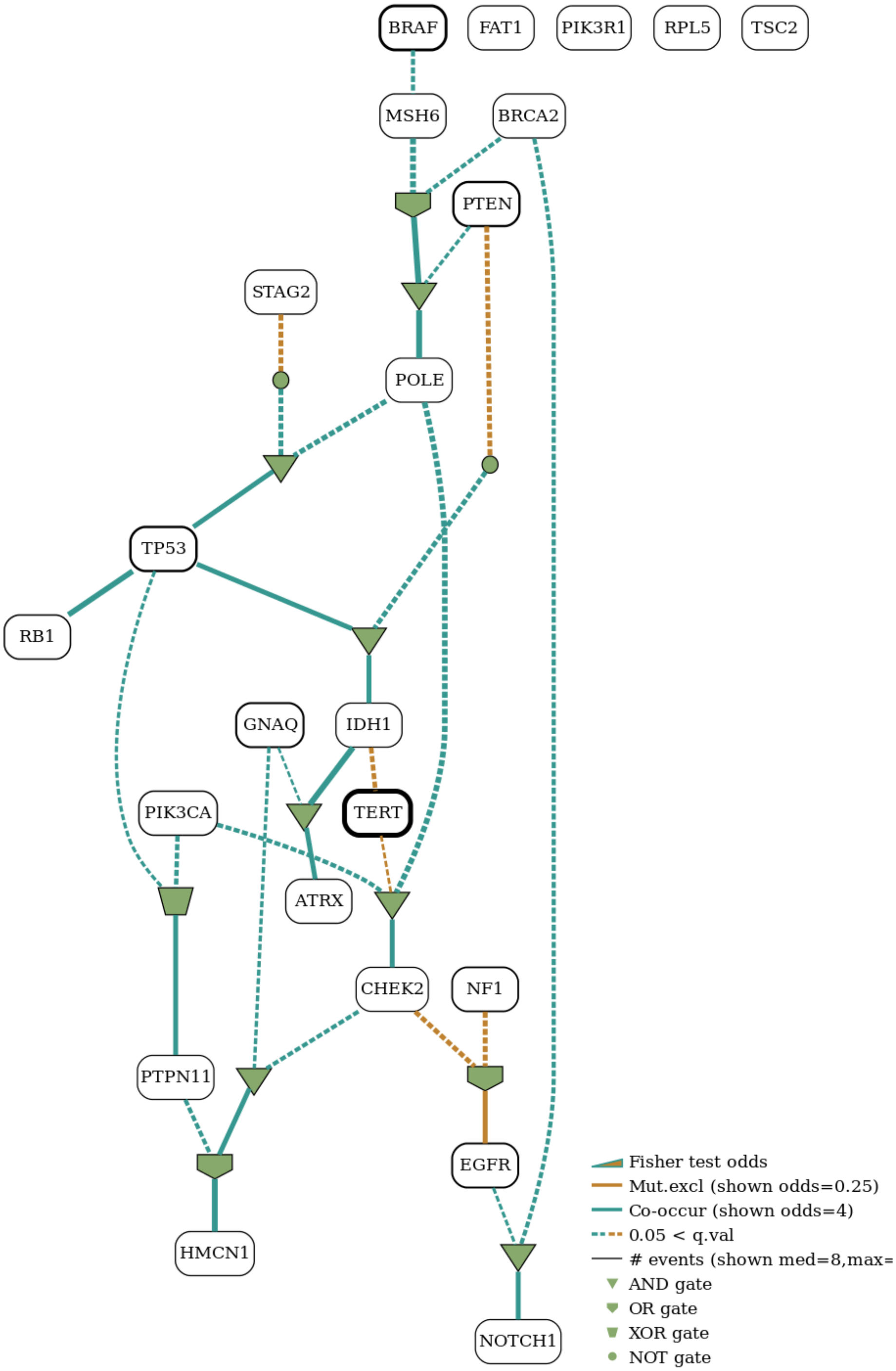

Supplementary Figure 11: Gated BN for the glioblastoma dataset (μ =5, ϵ =1).

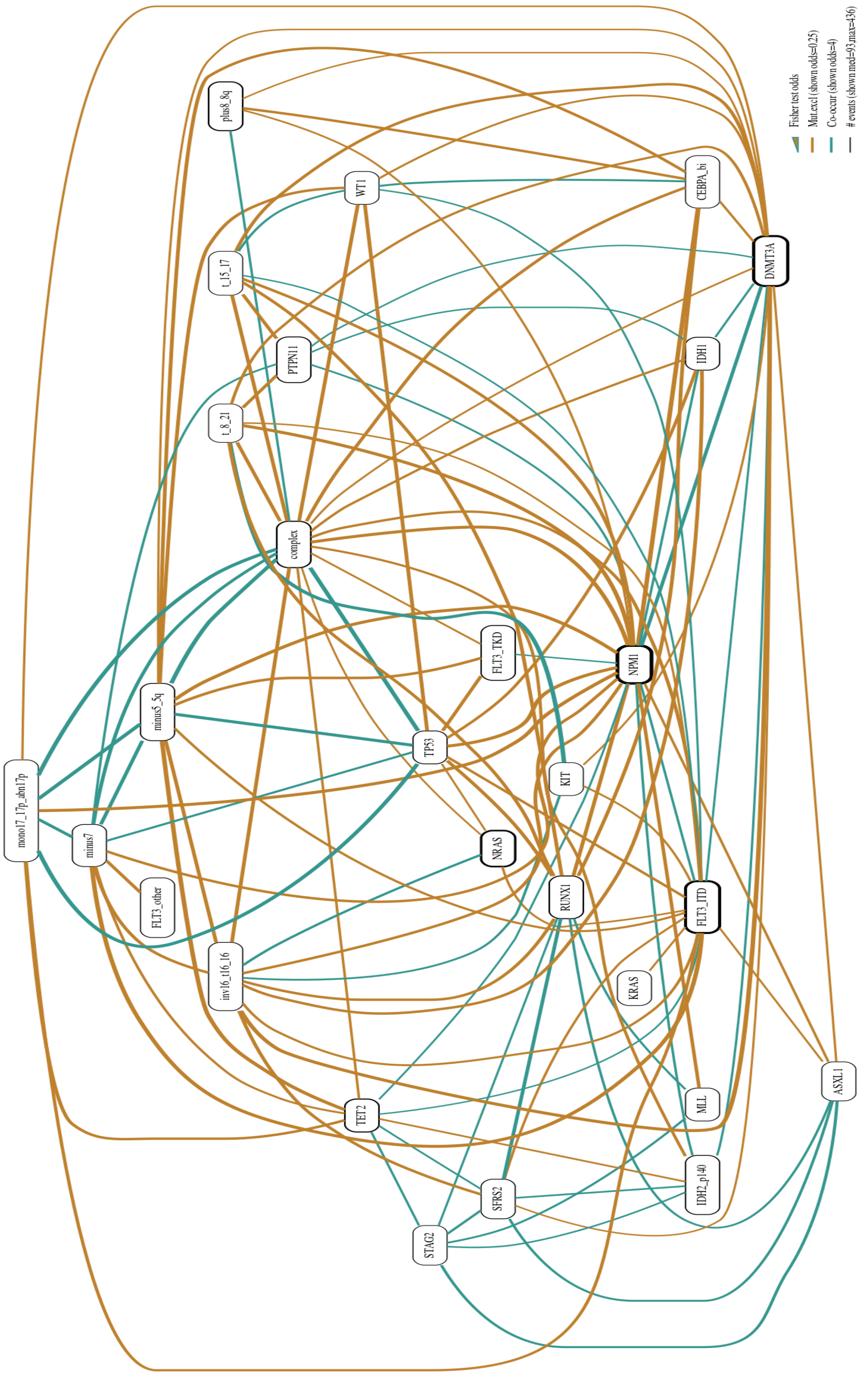

Supplementary Figure 12: Network for the AML dataset constructed by adding all edges for which the Fischer exact test (*R*'s fisher.test() function) between the two connected vertices returned a significant value (< 0.05).

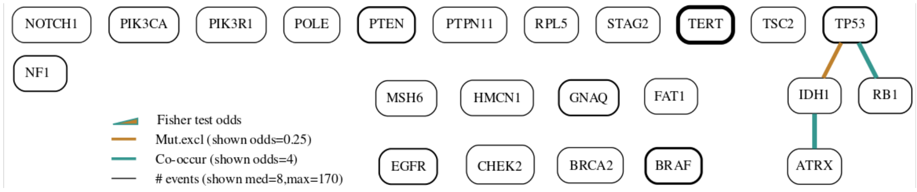

Supplementary Figure 13: Network for the glioblastoma dataset constructed by adding all edges for which the Fischer exact test (R's fisher.test() function) between the two connected vertices returned a significant value (< 0.05).

Supplementary Note 6: Familial heatmaps

For each of the datasets analysed and BN shown in main paper and the supplement we present their familial heatmaps. Each plot is a multi-part heatmap containing one element for each node in the network, with each heatmap showing value variation for the node and its parents.

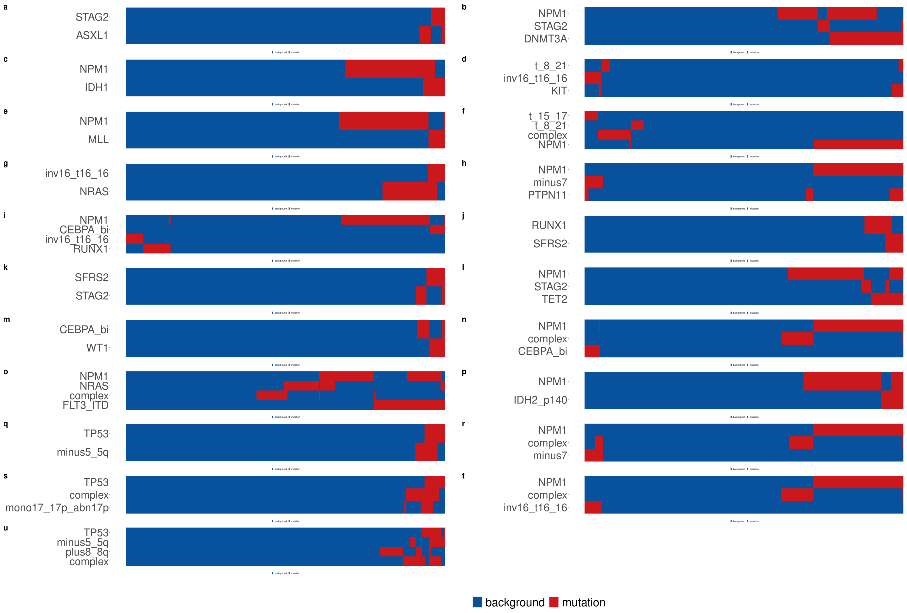

Supplementary Figure 14: Familial heatmaps for AML. Blue colour is used for non-events and red for a driver event be present. X-axis plots patients while Y-axis plots driver events. The events at each sub-plot correspond to a family in the corresponding Bayesian network.

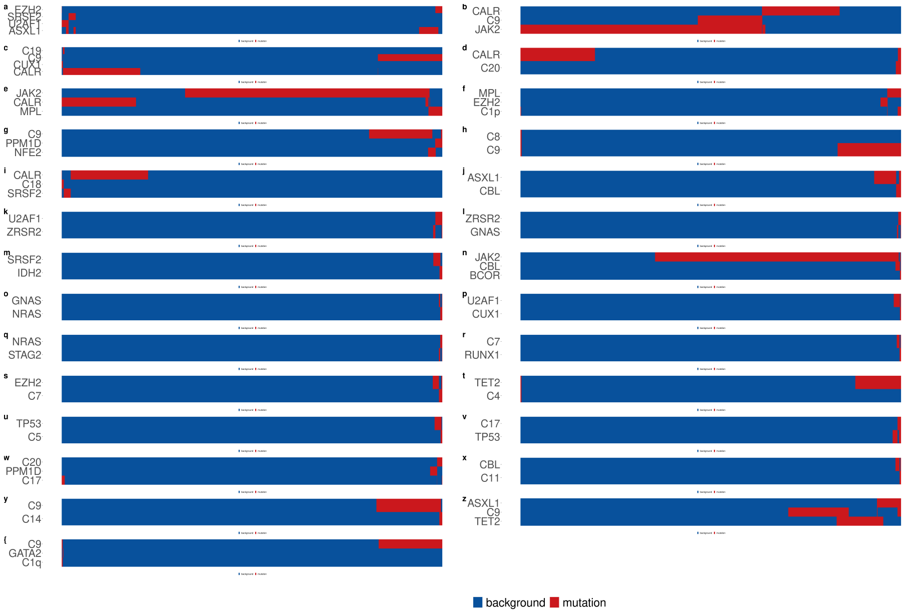

Supplementary Figure 15: Familial heatmaps for MPN. Blue colour is used for non-events and red for a driver event be present. X-axis plots patients while Y-axis plots driver events. The events at each sub-plot correspond to a family in the corresponding Bayesian network.

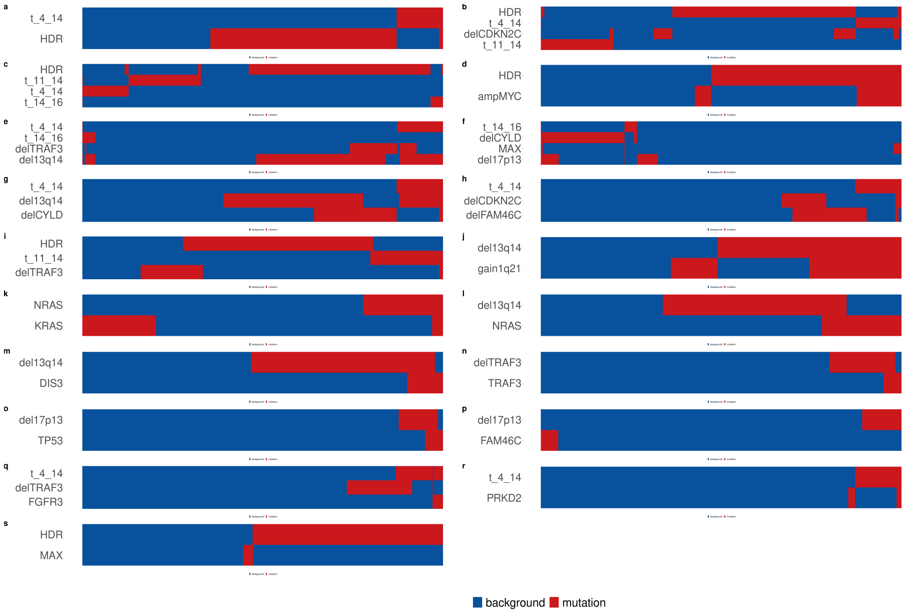

Supplementary Figure 16: Familial heatmaps for myeloma. Blue colour is used for non-events and red for a driver event be present. X-axis plots patients while Y-axis plots driver events. The events at each sub-plot correspond to a family in the corresponding Bayesian network.

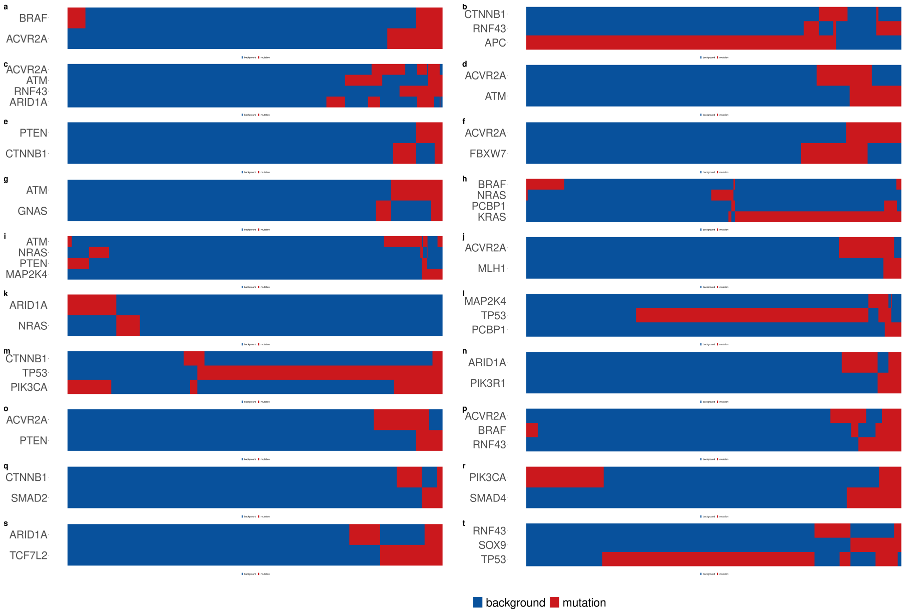

Supplementary Figure 17: Familial heatmaps for TCGA/COAD. Blue colour is used for non-events and red for a driver event be present. X-axis plots patients while Y-axis plots driver events. The events at each sub-plot correspond to a family in the corresponding Bayesian network.

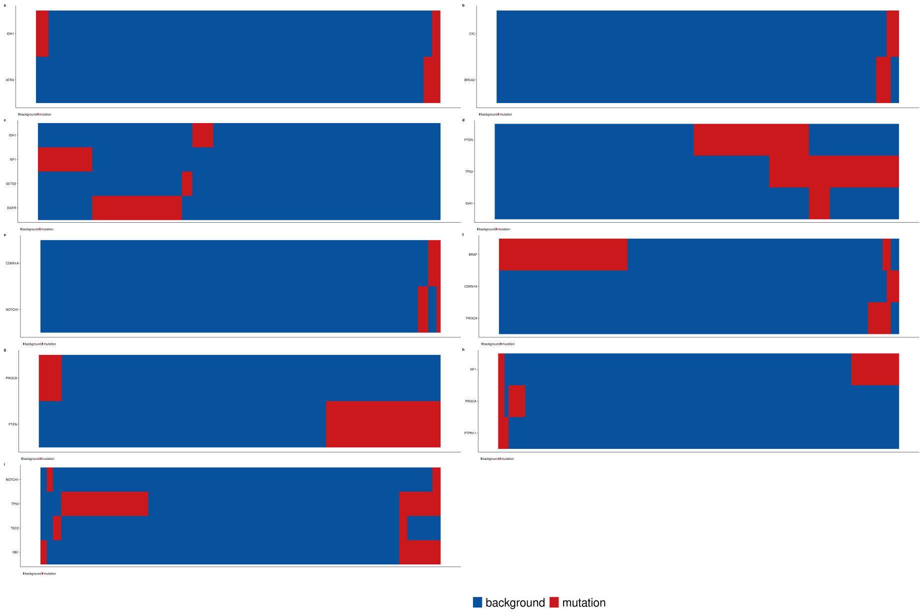

Supplementary Figure 18: Familial heatmaps for the glioblastoma dataset. Blue colour is used for non-events and red for a driver event be present. X-axis plots patients while Y-axis plots driver events. The events at each sub-plot correspond to a family in the corresponding Bayesian network.

Supplementary References

[1] N. Angelopoulos, S. Abdallah, and G. Giamas. Advances in integrative statistics for logic programming. *International Journal of Approximate Reasoning*, 78:103–115, November 2016.

[2] M. Bartlett and J. Cussens. Integer linear programming for the Bayesian network structure learning problem. *Artificial Intelligence*, 244:258 – 271, 2017.

[3] G. Gamrath, D. Anderson, K. Bestuzheva, W.-K. Chen, L. Eifler, M. Gasse, P. Gemander, A. Gleixner, L. Gottwald, K. Halbig, G. Hendel, C. Hojny, T. Koch, P. Le Bodic, S. J. Maher, F. Matter, M. Miltenberger, E. Mühmer, B. Müller, M. E. Pfetsch, F. Schlösser, F. Serrano, Y. Shinano, C. Tawfik, S. Vigerske, F. Wegscheider, D. Weninger, and J. Witzig. The SCIP Optimization Suite 7.0. ZIB-Report 20-10, Zuse Institute Berlin, March 2020.

[4] J. Wielemaker, T. Schrijvers, M. Triska, and T. Lager. SWI-Prolog. *Theory and Practice of Logic Programming*, 12(1-2):67–96, 2012.
