# Supplementary material for: Bayesian networks elucidate complex genomic landscapes in cancer
Source: Commun Biol. 2022 Apr 4;5:306. doi: 10.1038/s42003-022-03243-w (PMC8980036; doi:10.1038/s42003-022-03243-w)
Supplement: Supplementary file 3 — Description of Additional Supplementary Files [file 42003_2022_3243_MOESM3_ESM.pdf]

Description of Additional Supplementary Files

File name: Supplementary Data 1
Description: All input matrices to learning the BNs in this paper.
